# Performance Degradation and Microscopic Analysis of Lightweight Aggregate Concrete after Exposure to High Temperature

**DOI:** 10.3390/ma13071566

**Published:** 2020-03-28

**Authors:** Weijing Yao, Jianyong Pang, Yushan Liu

**Affiliations:** 1 School of Civil Engineering and Architecture, Anhui University of Science and Technology, Huainan 232001, China; pangjyong@163.com (J.P.); liuyushan1997@126.com (Y.L.); 2 Engineering Research Centre of Underground Mine Construction, Ministry of Education, Huainan 232001, China; 3 State Key Laboratory of Mining Response and Disaster Prevention and Control in Deep Mine, Anhui University of Science and Technology, Huainan 232001, China

**Keywords:** lightweight shale ceramsite concrete, high temperature, ultrasonic testing, performance deterioration, microstructure

## Abstract

This study analyses the deterioration of mechanical properties in lightweight concrete after exposure to room temperature (20 °C) and high temperature, i.e., up to 1000 °C, including changes in visual appearance, loss of mass, and compressive strength. All-lightweight shale ceramsite aggregate concrete (ALWAC) and semi-lightweight shale ceramsite aggregate concrete (SLWAC) are prepared using an absolute volume method to analyse the relationships between relative ultrasonic pulse velocity, loss rate of compressive strength, damage degree, and temperature levels. Our results show that, under high temperature, the lightweight aggregate ceramsite concrete performs better compared to normal concrete. After exposure to 1000 °C, the ALWAC shows a strength loss of no more than 80%, while the normal concrete loses its bearing capacity, with a similar strength loss as the SLWAC. Furthermore, the relative ultrasonic pulse velocity and damage degree are used to evaluate the effects of high temperature on the concretes, including the voids and cracks on the surface and inside of the specimens, which induces the deterioration of mechanical properties and contributes to the thermal decomposition of the cementing system and the loss of cohesion at the aggregate interface. Based on internal structure analyses, the results from this study confirm that the lightweight aggregate concrete shows a high residual compressive strength after exposure to the high temperature.

## 1. Introduction

Lightweight aggregate, mixed with concrete to form lightweight aggregate concrete, has been widely used in building insulation walls, tall buildings, and bridge construction [1,2]. Compared to the normal aggregate, the lightweight aggregate has the characteristics of lower weight, porous surface, and strong water absorption, providing lower bulk density and large moisture content, which not only achieves the internal curing effect and enhances the interface structure density, but also reduces the occurrence of burst in the fire. Ceramsite is a kind of lightweight porous aggregate, added into concrete. Currently, shale ceramsite lightweight aggregate concrete has been broadly used as the lightweight aggregate concrete [3,4]. However, when concrete structures are subjected to high temperature disasters, such as fire, concrete can deteriorate for a variety of reasons. Indeed, exposure to fire and high temperature often cause serious deterioration of concrete performance. Therefore, manufacturing high-heat-resistant concrete with high performance is considered as an important direction for future development [5,6]. This study is motivated by the laboratory test method to reveal the degradation process of lightweight shale ceramsite concrete from room temperature (about 20 °C), to the high temperature, up to 1000 °C with an interval of 100 °C, which is used to simulate the deterioration process of concrete in a high temperature environment, subjected to fire in a short time (usually 2–3 h).

The high temperature characteristics of ceramsite concrete have been widely researched. For instance, Zhou et al. analysed the residual strength, appearance characteristics, and microstructure of all-lightweight concrete after experiencing high temperature [7]. Jiang et al. designed a series of tests to analyse the high temperature characteristics of shale ceramsite with different mixing ratios, at different temperatures [8]. He [9] and Guo et al. [10] suggested that the high temperature burst resistance of the lightweight aggregate concrete was improved by applying an aggregate surface covered treatment. Yoon et al. validated the excellent performance of concrete which consists of a clay-ash-based artificial lightweight aggregate by comparing its high temperature properties against granite-based aggregate concrete [11]. Go et al. studied the fire-resistance properties of a reinforced lightweight aggregate concrete wall and concluded the lightweight aggregate walls maintained a superior structure behaviour after exposure to the high temperature [12]. Current literature review suggests that the lightweight aggregate concrete can maintain a complete structure after exposing to the high temperature, while, the internal structure suffers from different degrees of deterioration. However, the quantitative methods have not been used to indicate the internal damage degree of deterioration. Currently, ultrasonic non-destructive testing method is considered as a convenient, fast, reliable, and reproducible method for evaluating internal damage [13,14]. However, so far, limited studies have focused on the application of the ultrasonic method for evaluating the performance of lightweight aggregate concrete after exposure to the high temperature.

Furthermore, in recent studies, many modern laboratory methods have been used to reveal concrete deterioration mechanism after exposure to high temperature [15,16]. For instance, X-ray computed tomography (X-ray CT) and scanning electron microscopy (SEM) are applied to observe the internal structure of concrete specimens after exposure to high temperature, which can disclose fractal characteristics of fragmentation changing with temperature [17]. Moreover, SEM and energy dispersive spectrometer (EDS) analysis are performed to analyse internal structure properties of lightweight concrete after exposed to high temperatures [18]. In addition, nuclear magnetic resonance and SEM techniques are combined to observe the spectrum distribution, pore size distribution, and flaw development of concrete after exposure to high temperature [19]. A current literature review suggests that, at present, SEM has been widely used to study the microstructure of concrete. Also, the microscopic composition of the interface between aggregate and cementitious materials as well as the interaction between components are considered as significant factors to further improve the performance of materials [20]. The results from relative studies suggest new pores and channels appeared in the concrete, in response to the high temperature. Indeed, as the temperature increased, cement paste and hydration products became looser and adhesion between the aggregate and slurry weakened, causing occurrence of macroscopic crack [21,22,23,24]. However, the mechanisms behind the deterioration of concrete performance, internal damages, and microstructure changes after exposure to high temperature have not been studied in depth. The lightweight aggregate concrete is significantly different from normal concrete because the strong water absorbing force of the lightweight aggregate may help to reduce the porosity and increase the micro-hardness of the interfacial transition zone [25,26]. So far, few studies have focused on the change of the interfacial transition zone in the concrete, after exposure to high temperature. Therefore, the current study focuses on the relationship between the mechanical properties deterioration, damage degree, and microstructure changes of the lightweight aggregate concrete.

This study aims to (1) investigate the performance of all-lightweight and semi-lightweight shale ceramsite concrete under high temperature, (2) analyse the strength loss rate of the lightweight aggregate concrete after exposure to various temperatures, (3) investigate the microstructural changes of the interfacial transition zone, using SEM, and (4) explore the mechanism of degradation of the lightweight aggregate concrete after exposure to high temperature.

## 2. Materials and Methods

### 2.1. Raw Materials

Cement and fly ash are used as cementitious materials. The cement, used in this study, is made of Huainan Bagongshan P·C 42.5 composite Portland cement (Huainan, China), with 3-day and 28-day compressive strength of 29.9 MPa and 47.7 MPa, respectively. The fly ash is Grade Ⅰ, produced by Huainan Pingwei Power Plant (Huainan, China). Furthermore, fine sand is used as a fine aggregate, with a fineness modulus of 2.8. Coarse aggregate, used in this study, includes the lightweight aggregate and limestone gravel, both with a continuous gradation of 5–15 mm. The lightweight aggregate is shale ceramsite, produced by Anhui Jinrui Building Materials (Huainan, China). The basic performance parameters of the shale ceramsite are presented in Table 1. Moreover, the water-reducing admixture, used in this study, is produced by Shanxi Qinfen Building Materials Factory (Weinan, China).

**Figure 1:**
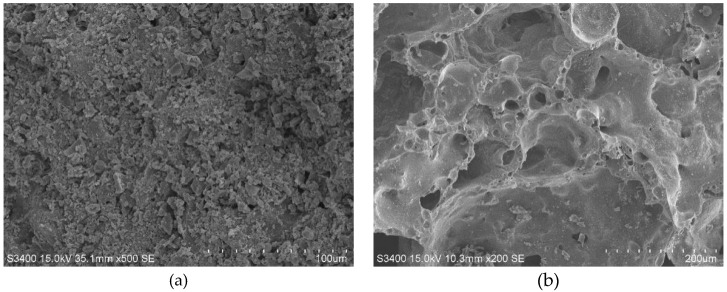
Micro-structures of shale ceramsite: (**a**) Shell, (**b**) Core.

Shale ceramsite is a kind of artificial porous lightweight aggregate, in which the outer surface has a coarse ceramic structure, with visible open pores and cracks [27]. After dissection, the core has a large aperture with spherical holes, forming a passage with a clear network structure (Figure 1). Because of this special structure, some cement paste penetrates the lightweight aggregate, during the fresh mixing stage. During the hardening stage, the cement hydration and the relative humidity inside the material are reduced, and the moisture stored inside the ceramsite is released to internally maintain the surrounding cement paste and the surrounding cement matrix becomes denser and more uniform [28].

### 2.2. Mix Design

According to JGJ55-2011 “Normal Concrete Mix Design Specification” [29] and JGJ51-2002 “Lightweight Aggregate Concrete Technical Regulations” in China [30], the absolute volume method is used to design all-lightweight and semi-lightweight aggregate concretes. The volume sand rate is 40% and five types of concrete are designed, including NC, LC-100, LC-75, LC-50, and LC-25. The NC is normal concrete, while the LC-100 represents all-lightweight shale ceramsite aggregate concrete. The LC-75, LC-50, and LC-25 are the semi-lightweight shale ceramsite aggregate concrete in which the ceramsite, with a volume 75%, 50%, and 25%, respectively, is used instead of coarse aggregate gravel. Following Kong and Yu [25], before the preparation of concrete, the ceramsite is treated with water absorption for 1 h, which can form internal curing effect. The concrete mix ratio of each group is presented in Table 2 and the basic performance test results are demonstrated in Table 3.

As shown in Table 3, with the increase of ceramsite replacement rate, the concrete strength, apparent density, thermal conductivity, slump, and slump flow decrease to some extent. Meanwhile, the moisture content improves, reflecting the internal curing effect of ceramsite pre-wetting. When the replacement rate exceeds 50%, the apparent density meets the requirements of lightweight concrete below 1950 kg/m^3^, the thermal conductivity of the LC-100 is 42.4% lower than that of the NC, and the compressive strength reduces by 63.4%.

### 2.3. Experimental Design

**Figure 2:**
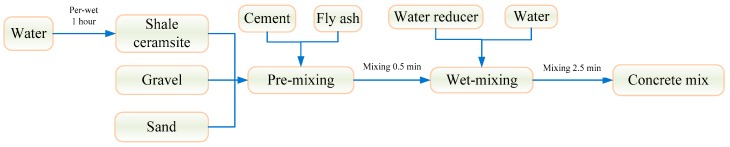
Forming process of concrete.

Following Kong and Yu [25], the lightweight aggregate was soaked in water for 1 h, before the preparation of concrete (Figure 2). Concrete test specimens with size of 100 mm × 100 mm × 100 mm (L × W × H) were cured for 28 days, under curing conditions with relative humidity of ≥95% and temperature of 20 ± 2 °C [31].

**Figure 3:**
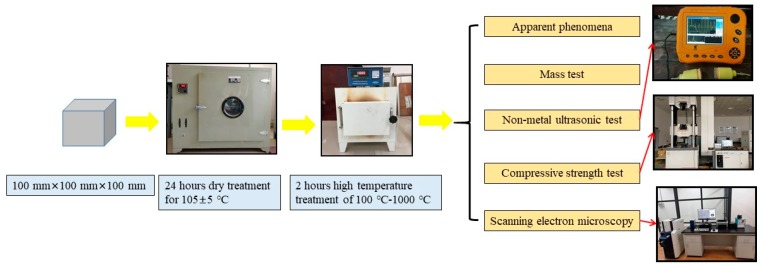
Test procedure.

Before the high temperature treatment, all the test specimens were dried in a drying baker at 105 ± 5 °C for 24 h to eliminate the influence of moisture content and to avoid excessive moisture content causing burst during heating [31]. The test specimens were heated using a chamber electric resistance furnace, with target temperature of 100, 200, 300, 400, 500, 600, 700, 800, 900, and 1000 °C, and a heating rate of 10 to 15 °C/min. Reaching to the target temperature, the temperature was kept constant for 2 h to ensure achieving the same temperature for the furnace and the internal parts of test specimen. When the temperature of furnace dropped to about 100 °C, the specimens were taken out and left for 48 h, before measuring various properties [8,9,10,11]. Subsequently, after each temperature, the ultrasonic pulse velocity of the specimens was measured using a non-metal ultrasonic test meter (NM-4B, Beijing Koncrete Engineering Testing Technology Company, Beijing, China). Two relative measuring points were arranged in each specimen to calculate the mean value [32]. Ultrasonic frequency was set up to 50 kHz and the emission voltage was 500 V, with the sampling period of 0.4 µs. After the ultrasonic measurement, the compression failure test (CSS-YAN3000, Changchun Testing Machine Research Institute, Changchun, China) was carried out and the relative residual compressive strength was calculated based on the ratio of the compressive strength of each specimen after high temperature to the dryness. Finally, the samples were selected from the crushed specimen to analyse the microscopic morphology of the interfacial transition zone (S-3400N, Hitachi Limited, Tokyo, Japan), by evaluating the joints of cement matrix, the lightweight aggregate, and cement matrix. Figure 3 represents the schematic diagram of the test procedure, including the drying baker (HK, Qinzhuo Environmental Equipment Testing Company, Dongguan, China) and chamber electric resistance furnace (KSW, Beijing Kewei Yongxing Instrument Company, Beijing, China).

## 3. Results and Discussion

### 3.1. Visual Appearance after High Temperature

**Figure 4:**
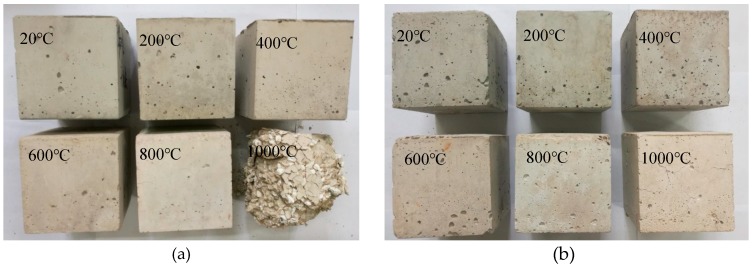
Appearance changes of concrete specimens after exposure to high temperature: (**a**) NC; (**b**) LC-100.

Figure 4 demonstrates the visual appearance changes in the NC and LC-100 specimens after exposure to the high temperature. As can be observed in the figure, below 800 °C, similar changes are observed in the appearance of test specimens. As the temperature increases, the colours of each group of samples gradually convert from blue-grey to light grey and become cream-coloured at 1000 °C (Figure 4). Between 20 and 300 °C, no significant changes occur in the specimens’ appearance. At this stage, the samples have a crisp knocking sound and no crack is observed over their surface. Between 300 and 500 °C, the specimens’ colours darken, showing a shade of brownish colour, with a gentle knocking sound. After 600 °C, obvious cracks begin to appear, also, the specimens’ colours change to greyish-white, with a low knocking sound. At 800 °C, the specimens’ colours become yellowish-white and continuous long cracks are observed over the samples’ surfaces. These results are consistent with those ofJiang et al. [8], He et al. [9], Guo et al. [10], Gong et al. [13,32], and Ma et al. [33].

However, after 900 °C and above, coarse crack, peeling, and scaling are observed only in the NC specimen. Subsequently, after 48 h, the internal cracks penetrate the surface, resulting in a severe break of the specimen (Figure 4a). Moreover, the sample has an overall loose structure (Figure 4a). In contrast, the all-lightweight ceramsite aggregate concrete, i.e., the LC-100, shows the advantage of high temperature resistance (Figure 4b). After 1000 °C, the integrity of the specimen is still good and there are obvious peeling, scaling, and surface crack. For semi-lightweight ceramsite aggregate concrete, with the reduction of ceramsite replacement rate, high temperature degradation gradually deepens, hence, similar effects are observed in samples LC-25 and NC. Indeed, gravel softened after being subjected to the high temperature, causing serious strength loss, high temperature degradation, and broken interface with the cement matrix [33,34].

### 3.2. Mass Loss after Exposure to High Temperature

**Figure 5:**
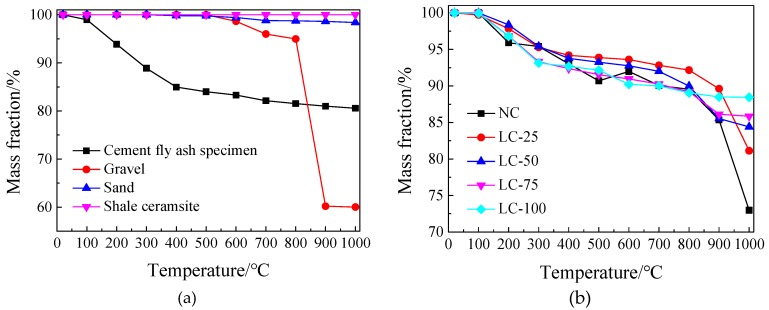
Mass loss of materials after exposure to high temperature: (**a**) Raw materials; (**b**) Concrete specimens.

The mass loss of each raw material and concrete specimens after exposure to different temperatures are presented in Figure 5.

#### 3.2.1. Raw Materials Mass Loss after Exposure to the High Temperature

The cement fly ash test specimen (10% fly ash content) shows a large mass loss rate below 600 °C, followed by a gentle decrease. The mass loss after exposure to 1000 °C is nearly 20% (Figure 5a). Since the main component of the cement after hydration is C-S-H (hydrated calcium silicate) gelation, below 600 °C, the crystallization water, free water, and C-S-H gelled adsorption water inside the test specimen are largely lost, resulted in a significant decrease in mass. Above 600 °C, the C-S-H begin to significantly decompose. However, the decomposition mass loss is small. Therefore, the mass loss rate of the cement fly ash sample decreases significantly with the increase of temperature and then stabilizes to 80% (Figure 5a).

The mass of gravel remains unchanged below 600 °C. Between 600 and 800 °C, the mass loss is about 5%. Above 900 °C, the mass loss decreases sharply, reaching 40% after exposure to 1000 °C (Figure 5a). After exposure to high temperature, the mass loss of sand is small. Indeed, the main component of gravel is CaCO_3_, which decomposes into CaO and CO_2_ at 600 to 820 °C, resulting in a significant mass reduction [35].

**Figure 6:**
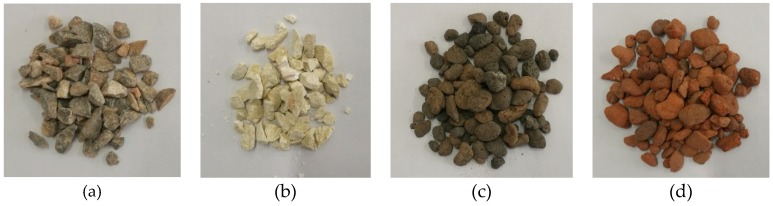
Appearance changes of gravel and ceramsite after exposure to high temperature of 1000 °C: (**a**) Gravel (20 °C); (**b**) Gravel (1000 °C); (**c**) Shale ceramsite (20 °C); (**d**) Shale ceramsite (1000 °C).

The shale ceramsite shows no mass loss after exposure to the high temperature. Indeed, the shale ceramsite is produced by high temperature sintering of shale, where the temperature of the sintering process exceeds 1000 °C. Comparing the appearance of gravel and shale ceramsite at the temperature of 1000 °C, the gravel’s colour changes from bluish-white to snow-white and the strength loss is considerable (Figure 6a,b). In contrast, the ceramsite changes from brown to red in color, while the sample is still intact and the strength loss is small, indicating high temperature resistance property of the ceramsite (Figure 6c,d).

#### 3.2.2. Concrete Specimens Mass Loss after Exposure to High Temperature

From Figure 5b, the mass loss trend of each concrete specimen is consistent, after exposure to high temperature. Mass loss is controlled in 5% and 10% below 200 °C and above 600 °C, respectively. For this temperature interval, the mass loss is mainly caused by the loss of cement binder and the mass further decreases after exceeding 600 °C. For the NC, the mass decreases significantly above 800 °C, and mass loss reaches 27.01% after exposure to 1000 °C. For the LC-100, the mass loss curve is gentle after exceeding 600 °C, reaching 11.56% after exposure to 1000 °C. For semi-lightweight ceramsite aggregate concrete, the mass loss gradually increases as the replacement rate decreases. After exposure to 1000 °C, the mass losses of the LC-75, LC-50, and LC-25 are 14.14%, 15.60%, and 18.87%, respectively. The latter suggests the high temperature resistance advantage of ceramsite, compared to gravel.

### 3.3. Strength Loss after Exposure to High Temperature

**Figure 7:**
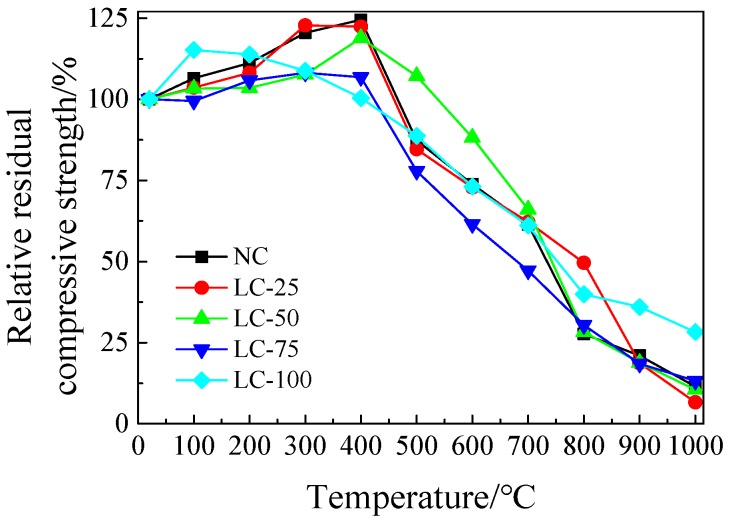
Relative residual compressive strength of concrete after exposure to high temperature.

The relative residual compressive strength of each concrete specimen after exposure to high temperature are presented in Figure 7. Below 400 °C, the relative residual compressive strength of various types of concrete changes, slightly. Moreover, under different degrees, from 100 °C to 400 °C, the strength increases, resulting in a maximum growth of about 20%. Indeed, the concrete is heated from normal temperature to a secondary hydration reaction of “high temperature curing” at 400 °C, which resulted in an increase in strength, similar to “steam curing” [36]. Water is evaporated from gel pores by high temperature heating, but the dense concrete structure makes water different for overflow. Furthermore, the crystallization water escapes inside the concrete and the concrete pore structure does not change significantly. As a result, the hydration with unhydrated cement paste continues to increase the strength of the cement matrix. These results are in line with the findings of recently conducted research in this field [37,38,39]. Above 500 °C, the compressive strength decreases sharply, because the internal pore structure of the concrete is severely roughened at 500–800 °C, the C-S-H gel is severely dehydrated, losing the effect of cementation. Therefore, the crack expands and penetrates by the cement shrinkage and gravel softening and expansion. These changes are also reflected in the microscopic analyses (see Section 3.6). After experiencing a high temperature of 900–1000 °C, the compressive strength and bearing capacity are greatly lost.

In addition, as it can be seen from Figure 7, the strength loss rate of the NC after treatment at 1000 °C is 88.5%, while the LC-100 shows a strength loss rate of 71.7%, after experiencing a high temperature of 1000 °C. The reason is that the performance of shale ceramsite changes slightly, after exposure to high temperature and the strength loss of the lightweight aggregate concrete is mainly caused by high temperature damage of the cement gel. For the samples LC-75, LC-50, and LC-25, similar to the NC, when the temperature is lower than 400 °C, the strength increases slightly. After exposure to the high temperature of 500–1000 °C, the test specimen shows a serious strength loss. Indeed, the semi-lightweight ceramsite aggregate concrete contains both calcareous gravel and shale ceramsite. The calcareous gravel and cement gel are both decomposed by high temperature destruction, resulting in a loss of cementation and affecting the overall strength.

### 3.4. Internal Damage after Exposure to High Temperature

Ultrasonic testing is performed to evaluate the internal damage of concrete specimens after exposure to high temperature. Following Gong et al. (2018) and Gong and Zhang (2019), the relative ultrasonic pulse velocity and damage degree are used as evaluation parameters to eliminate the influence of aggregate type, particle size, cement type, and dosage on the ultrasonic propagation characteristics [32,40], as follows:

(1) $$v_{R}=v_{T}/v_{0}$$

(2) $$D=1-{\left(v_{T}/v_{0}\right)}^{2}$$

where, *v_R_* is relative ultrasonic pulse velocity, *v_T_* is ultrasonic pulse velocity after exposure to high temperature (m/s), *v*_0_ is ultrasonic pulse velocity before exposure to high temperature (m/s), and *D* is damage degree.

**Figure 8:**
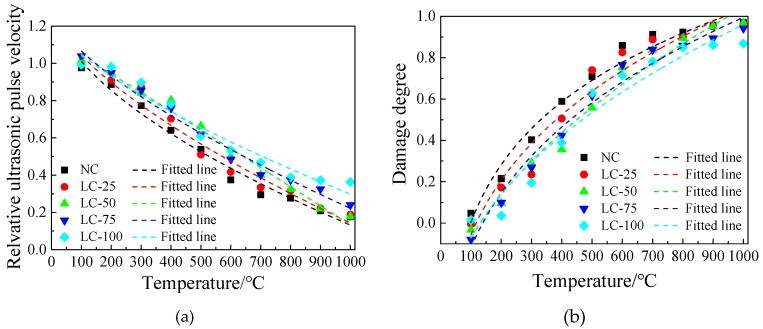
Relationship between ultrasonic parameters and temperature of concrete: (**a**) Relationship between relative ultrasonic pulse velocity and temperature; (**b**) Relationship between damage degree and temperature.

Figure 8 represents the processed data. The regression fitting was performed using a suitable function and the fitting results are shown in Table 4. Based on our results, the two ultrasonic parameters of the main frequency and the amplitude of the concrete specimens after exposure to different temperatures are somehow similar, indicating that these two types parameters are unsuitable for evaluating and analysing the performance changes of concrete after exposure to high temperature. This is consistent with the conclusions of the relevant research [32,40,41].

As can be seen in Figure 8, the relative ultrasonic pulse velocity and damage degree of each concrete specimen have a good correlation with the heating temperature. As the heating temperature increases, the relative ultrasonic pulse velocity gradually decreases, and damage degree increases continuously, indicating that the internal damage of the test specimen accumulates at high temperatures [14]. It can be seen from the fitting results that the fitting effect of each concrete is good, indicating that it is reasonable and feasible to evaluate the internal damage of concrete after exposure to high temperature using ultrasonic parameters (Table 4). In addition, with the ceramsite addition decreasing of LC-100, LC-75, LC-50, and LC-25, the fitting formula is closer to NC.

### 3.5. The Relationship between Internal Damage and Strength Loss

According to Figure 7, the concrete specimens show a slight increase in strength due to “high temperature curing” within 400 °C and the ultrasonic propagation is affected by many factors. The relative ultrasonic pulse velocity and damage degree continue to increase with the heating temperature, which fails to reflect this change. Therefore, discussing the relationship between relative ultrasonic pulse velocity, damage degree, and strength loss, we do not consider the impact of this segment. The fitting effect is optimal after deducting the temperature range of 100–200 °C.

**Figure 9:**
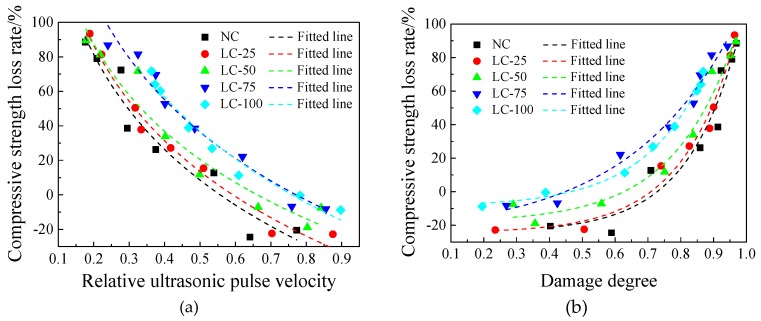
Relationship between ultrasonic parameters and compressive strength loss rate of concrete after exposure to high temperature: (**a**) Relationship between relative ultrasonic pulse velocity and compressive strength loss rate; (**b**) Relationship between damage degree and compressive strength loss rate.

Figure 9 shows the relationship between the relative ultrasonic pulse velocity, damage degree, and the compressive strength loss rate of each concrete specimen. The appropriate functions are used for regression analysis (Table 5). Based on the results, the correlation coefficient R^2^ is greater than 0.90, and the fitting result is good, indicating that the non-destructive testing method is feasible to evaluate the compressive strength loss rate with the relative ultrasonic pulse and damage degree. In addition, similar to the fitting relationship between the ultrasonic parameters and heating temperature, with decreasing ceramsite content in the semi-lightweight aggregate concrete, the fitting relationship is closer to the NC.

### 3.6. Microscopic Analysis

#### 3.6.1. Analysis of High Temperature Degradation Process of Cement Paste

**Figure 10:**
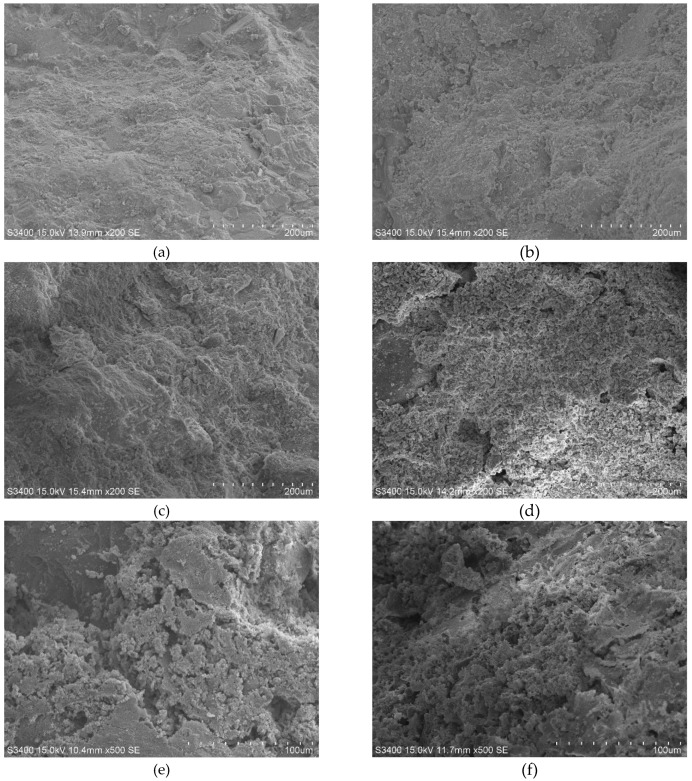
Micro-structures of cement matrix of NC after exposure to different temperature: (**a**) 20 °C, (**b**) 200 °C, (**c**) 400 °C, (**d**) 600 °C, (**e**) 800 °C, (**f**) 1000 °C.

Figure 10 demonstrates the microstructure of the NC cement paste after exposure to different temperatures. Under normal temperature, the cement hydration paste is tightly combined, the structure is complete and different phases are dense and continuous. Furthermore, the crystal water and a large amount of gel are existed at this stage. After exposing to temperature of 200–400 °C, no obvious changes in the microstructure are observed. However, the internal free water and capillary water vaporize and evaporate. Moreover, the C-S-H gel adsorption water is lost, promoting cement hydration reaction to form “high temperature curing”. Hence, the compressive strength is improved. Above 600 °C, a large amount of water is lost and lots of pores appear on the gelled surface. Furthermore, the gel is decomposed, the structure of the cement paste is destroyed, the structure is loose and slack. At this temperature, the macroscopic performance shows whitening of the specimen and cracks appear on the surface. At 800–1000 °C, further decomposition and generation of loose and porous materials result in the appearance of a large number of long and wide cracks on the surface of the specimens. After 48 h, the specimens are damaged and peeled off and a large amount of coarse aggregate are peeled off from the cement paste, and the interface adhesion force is attenuated or even lost. The compressive strength decreases by more than 70% after treatment of 800 °C and the bearing capacity is basically lost after exposure to 1000 °C [42,43].

#### 3.6.2. Analysis of High Temperature Degradation in Transition Zone of the Lightweight Aggregate Concrete

**Figure 11:**
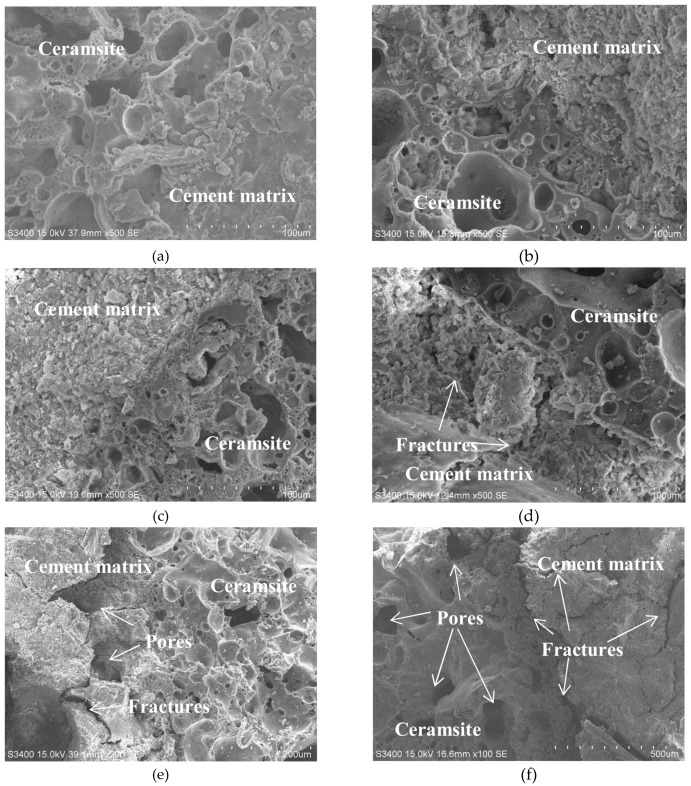
Micro-structures of interfacial transition zone of LC-100 after exposure to different temperature: (**a**) 20 °C, (**b**) 200 °C, (**c**) 400 °C, (**d**) 600 °C, (**e**) 800 °C, (**f**) 1000 °C.

Figure 11 shows the evolution process of high temperature degradation in the interface transition zone of the LC-100. At normal temperature, the ceramsite is bonded with cement matrix, the cement matrix structure is dense, and no defects, such as cracks and holes, are existed over the surfaces. However, above 400 °C, a great water loss occurs in the interface zone, causing the stratification and looseness of the cement paste. However, no obvious signs of hole defects are observed and the bonding between ceramsite and cement matrix remains very strong. The mechanical meshing structure is undamaged and the macroscopic performance is slightly improved. The compressive strength of the specimen increases by 13.8% at 200 °C. Above 600 °C, the cement paste is decomposed and destroyed, resulting in appearance of obvious cracks, holes, and interpenetration. After exposure to 800–1000 °C, the cement paste is loose and the adhesion to the ceramsite is weakened or even disappeared, resulting in a serious loss of macro strength. The strength loss was 60.1% at 800 °C and 71.7% at 1000 °C.

**Figure 12:**
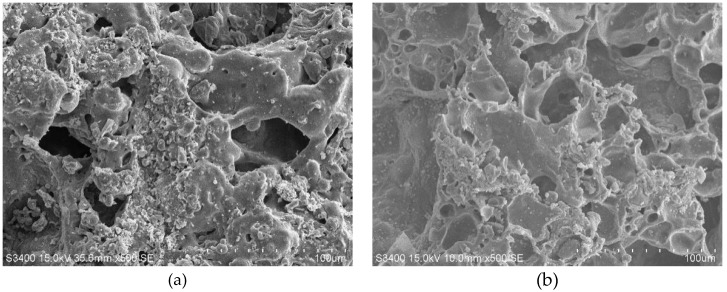
Micro-structures of shale ceramsite after exposure to 1000 °C: (**a**) Shell; (**b**) Core.

Ceramsite is an excellent high temperature resistance material. Microscopic characteristics of shell and core at the room temperature (i.e., 20 °C) (Figure 1) and after treatment of 1000 °C (Figure 12) suggests that after exposure to high temperature, some defects, such as holes, are observed in the porous ceramic material in the outer shell, however, the internal structure remains intact (Figure 12). Therefore, the strength loss of the lightweight aggregate concrete after exposure to high temperature is mainly due to the weakening of cement paste and the loss of interfacial adhesion. Because of the integrity of ceramsite, no softening and decomposition of calcareous aggregate occur in the samples. In the macroscopic view, the specimen is complete, with no peeling and lack of corners. This also explains why the high-temperature strength loss of the lightweight aggregate concrete is better compared to the NC.

## 4. Conclusions

Compared to the normal concrete, the all-lightweight shale ceramsite aggregate concrete has a great advantage, due to its high temperature performance. Based on our experimental research, after exposure to 1000 °C, the all-lightweight shale ceramsite aggregate concrete specimen remains intact, without any damage and peeling. Under this temperature, the mass and compressive strength loss are 11.56% and 71.7%, respectively, while under the same temperature, these values for the normal concrete are 27.01% and 88.5%, respectively. Furthermore, the strength loss rate of the semi-lightweight shale ceramsite aggregate concrete is similar to the normal concrete, because of the multiple effects of high temperature softening of calcareous gravel, high temperature decomposition, and decomposition of the cement paste. In contrast, the ceramsite lightweight aggregate is an excellent high temperature resistant material, resulting in a limited loss of mass and strength after exposure to the high temperature.

A comparative analysis of the relationship between relative ultrasonic pulse velocity, damage degree, heating temperature, and compressive strength loss rate suggest that with increasing the heating temperature, the relative ultrasonic pulse velocity decreases gradually, while the damage degree and the compressive strength loss rate increase. The fitting effect of the regression formula is good, suggesting that it is reasonable and feasible to use relative ultrasonic pulse velocity and damage degree as evaluation parameters to judge the performance degradation of concrete after exposure to high temperature.

Microscopic analysis of the interfacial transition zone between the cement paste and the lightweight aggregate after exposure to different temperatures show that as the temperature increases, the cement gel is loosened and dispersed from compaction and the bond strength with the aggregate is gradually lost, resulting in loss of macroscopic strength. However, the lightweight aggregate ceramsite is not significantly damaged after exposure to high temperature, suggesting its excellent high temperature resistant property, which is the main reason that the residual strength of the lightweight aggregate concrete is higher than that of normal concrete after exposure to the high temperature.

In addition, through experimental research, it can be concluded that there is an objective relationship between the deterioration of mechanical properties, damage degree, and microstructure changes of the lightweight aggregate concrete. After undergoing different degrees of high temperature, the cement hydrates are thermally decomposed, causing voids and cracks on the surface and inside of the concrete specimen, which is the primary reason for the deterioration of mechanical properties and the increase of damage degree.

## Figures and Tables

**Table 1:** Physical properties of shale ceramsite.

Performance	Particle Size (mm)	Bulk Density (kg/m^3^)	Apparent Density (kg/m^3^)	Cylinder Compressive Strength (MPa)	1 h Water Absorption (by mass) (%)	Shape	Silt Content (%)
Shale ceramsite	5–15	415	769	≥2.5	9.5–12.0	Ordinary type	≤1.6

**Table 2:** Mix proportions of concrete.

Number	Binding Material		Coarse Aggregate		Sand	Water	Water Reducer	Water Cement Ratio
	Cement	Fly Ash	Gravel	Shale Ceramsite				
NC	421	47	856.00	-	856.00	177.84	4.68	1:0.38
LC-25	421	47	835.06	77.92	702.54	168.48	4.68	1:0.36
LC-50	421	47	565.03	158.00	712.28	159.12	4.68	1:0.34
LC-75	421	47	286.37	240.24	722.01	149.76	4.68	1:0.32
LC-100	421	47	-	324.64	731.74	140.40	4.68	1:0.30

**Table 3:** Performances of different types of concrete.

Number	Workability		Compressive Strength (MPa)		28-day Apparent Density (kg/m^3^)	28-day Moisture Content (%)	28-day Thermal Conductivity (W/(m·K))
	Slump (mm)	Slump Flow (mm)	3-day	28-day			
NC	175	350	14.83	38.65	2272.00	1.10	1.65
LC-25	125	300	14.29	30.67	2167.30	2.64	1.54
LC-50	95	260	14.85	24.51	2056.48	3.01	1.32
LC-75	75	240	15.06	24.33	1913.00	4.30	1.15
LC-100	50	200	11.03	14.15	1679.33	5.29	0.95

**Table 4:** Fitting results of ultrasonic parameter and temperature of concrete.

Number	Relative Ultrasonic Pulse Velocity *v_R_*		Damage Degree *D*	
	Fitting Formula	*R* ^2^	Fitting Formula	*R* ^2^
NC	$v_{R}=-22.15\frac{T^{0.58}}{1000}+1.33$	0.98	*D* = 1.64*T*^0.13^ − 2.97	0.97
LC-25	$v_{R}=-11.75\frac{T^{0.66}}{1000}+1.29$	0.96	*D* = 0.39*T*^0.26^ − 1.38	0.94
LC-50	$v_{R}=-0.83\frac{T^{1.03}}{1000}+1.13$	0.98	*D* = 0.05*T*^0.51^ − 0.56	0.97
LC-75	$v_{R}=-8.83\frac{T^{0.69}}{1000}+1.28$	0.98	*D* = 0.34*T*^0.28^ − 1.35	0.97
LC-100	$v_{R}=-7.77\frac{T^{0.69}}{1000}+1.24$	0.95	*D* = 0.07*T*^0.45^ − 0.64	0.93

Note: *v_R_* represents relative ultrasonic pulse velocity; *D* represents damage degree; *T* represents temperature.

**Table 5:** Fitting results between ultrasonic parameter and compressive strength loss rate of concrete after exposure to high temperature.

Number	Relative Ultrasonic Pulse Velocity *v_R_*		Damage Degree *D*	
	Fitting formula	*R* ^2^	Fitting Formula	*R* ^2^
NC	*C_t_* = −79.49*lnv_R_* − 46.53	0.93	*C_t_* = 0.24*e*^6.32*D*^ − 24.36	0.92
LC-25	*C_t_* = −79.66*lnv_R_* − 41.65	0.98	*C_t_* = 0.35*e*^5.99*D*^ − 24.63	0.97
LC-50	*C_t_* = −73.20*lnv_R_* − 30.24	0.94	*C_t_* = 0.68*e*^5.27*D*^ − 18.34	0.96
LC-75	*C_t_* = −84.01*lnv_R_* − 21.81	0.96	*C_t_* = 6.51*e*^3.06*D*^ − 25.57	0.97
LC-100	*C_t_* = −88.15*lnv_R_* − 24.15	0.97	*C_t_* = 0.75*e*^5.35*D*^ − 9.00	0.99

Note: *v_R_* represents relative ultrasonic pulse velocity; *D* represents damage degree; *Ct* represents compressive strength loss rate.
